# Stem cells can form gap junctions with cardiac myocytes and exert pro-arrhythmic effects

**DOI:** 10.3389/fphys.2014.00419

**Published:** 2014-10-29

**Authors:** Nicoline W. Smit, Ruben Coronel

**Affiliations:** ^1^Department of Clinical and Experimental Cardiology, Heart Centre, Academic Medical Centre, University of AmsterdamAmsterdam, Netherlands; ^2^L'Institut de RYthmologie et modélisation Cardiaque, Université Bordeaux SegalenBordeaux, France

**Keywords:** stem cells, cardiomyocytes, electrotonic connections, arrhythmias, clinical trials

## Abstract

Stem cell therapy has been suggested to be a promising option for regeneration of injured myocardium, for example following a myocardial infarction. For clinical use cell-based therapies have to be safe and applicable and are aimed to renovate the architecture of the heart. Yet for functional and coordinated activity synchronized with the host myocardium stem cells have to be capable of forming electrical connections with resident cardiomyocytes. In this paper we discuss whether stem cells are capable of establishing functional electrotonic connections with cardiomyocytes and whether these may generate a risk for arrhythmias. Application of stem cells in the clinical setting with outcomes concerning arrhythmogenic safety and future perspectives will also briefly be touched upon.

## Introduction

Electrical remodeling and loss of working myocardium after a myocardial infarction (MI) remains a major health challenge (McMurray and Pfeffer, 2005). Both processes disrupt the coordinated activation and repolarization of the heart. A theoretical remedy would be to replace the injured tissue with new cardiomyocytes (CM) that are capable of integrating and forming a functional network with the resident cardiomyocytes.

Stem cells have an unlimited proliferation ability and are capable of differentiating into multiple cell types (Wagers and Weissman, 2004). These two properties explain why so much time and energy has been put in making cell therapy effective for regeneration of the myocardium. Yet for proper functional integration within the host's myocardium there are several prerequisites including safety (the absence of adverse effects), a suitable delivery mode and proper timing of delivery. Although many questions concerning stem cells and their mechanism of actions remain, numerous clinical trials have been performed, with varying results.

The Myoblast Autologous Grafting in Ischemic Cardiomyopathy (MAGIC) trial, a multicenter randomized double blind and placebo-controlled study, was the first human trial concerning regenerative stem cell therapy involving skeletal myoblasts. Patients (*n* = 90) with prior MI, left ventricular dysfunction [left ventricular ejection fraction (LVEF) < 35%] and indications for coronary surgery received intra-myocardial injections of autologous myoblasts (low dose: 4 × 10^8^ or high dose: 8 × 10^8^) or placebo solutions. No changes were seen at 6 months in either global or regional left ventricular (LV) function between placebo treated patients and those treated with autologous skeletal myoblast (Eisen, 2008; Menasche et al., 2008). Arrhythmias were documented throughout the 6 months, although there was a higher occurrence in the pooled treatment group this was not significantly different from the control group, most likely owing to the small number of subjects.

In the CADUCEUS trial, cardio-sphere derived stem cells (CDC) were used, which were infused (low dose: 12.5 × 10^6^ or high dose: 25 × 10^6^) into the coronary artery of patients with LV dysfunction at 1.5–3 months post MI. The study was designed to assess safety. At 6 and 12 months follow-up period, despite the reduction in scar size and increase in viable myocardium in the group receiving CDC, no differences in ejection fraction were seen between the control and intervention groups (Makkar et al., 2012; Pompilio et al., 2012). Similar to the MAGIC trial non-sustained ventricular tachycardia (VT) occurred numerically more often in the CDC treated group. However, this was not statistically significant, again due to the small number of patients (17 patients in CDC group, 8 control patients).

The investigators of the SCIPIO and BOOST trials used C-kit^+^ cardiac derived stem cells and autologous bone marrow stem cells, respectively, and did show functional improvements (Wollert et al., 2004; Bolli et al., 2011). The SCIPIO trial enrolled and randomized post MI patients with left ventricular dysfunction (LVEF ≤ 40%) before coronary artery bypass surgery into either the treatment (16 patients) or control group (7 patients) (Bolli et al., 2011). At 4 months post-surgery 16 patients received intracoronary infusion of stem cells (1 × 10^6^). At 6 months post infusion, global improvement in left ventricular ejection fraction (LVEF) by approximately 8.2 ± 2.0 LVEF units was seen. LVEF increased by 12.3 ± 2.0 LVEF units at 1 year post treatment (Bolli et al., 2011). In the BOOST trial, patients who had undergone successful percutaneous coronary intervention for (acute) ST-segment elevation MI were randomly assigned to the intracoronary bone-marrow transfer (30 patients) or to the control infusion (30 control patients). Patients showed increased global LVEF (about 6%) at 6 months post intracoronary infusion, which is similar to the SCIPIO trial. The effects observed in the BOOST trial, however, were not long-lasting and at 18 months and 5 years follow-up no difference was detected between the cell treated and non-cell treated groups. At 12 months post treatment the positive hemodynamic effects of the SCIPIO trial were maintained. However, that was the last follow-up point and hence it remains unknown whether the positive effects are long lasting or are only maintained up to 12 months post treatment as was seen in the BOOST trial. The SCIPIO trial investigators reported no occurrence of adverse arrhythmogenic events due to cell therapy such as (ventricular) arrhythmias but did not provide a definition of ventricular arrhythmias (Chugh et al., 2012). The BOOST trial reported that there was no difference in occurrence of adverse event, including arrhythmias, between treatment group and control group. However, also in this trial report, arrhythmias were not defined (e.g., duration of VT) and only mentioned as occurrence of VT (Meyer et al., 2006). In addition, similar to the MAGIC and CADUCEUS trial a limited number of patients were involved.

The clinical trials show that delivery of stem cells (either by intracoronary or intra-myocardial injection) is feasible and safe. However, whether the applied stem cells did differentiate and contribute to long-term functional improvements and safety remains questionable. The main reasons for this uncertainty are methodological differences between the trials and the low number of patients involved. Moreover, electrophysiological safety has not been extensively addressed and the electrophysiological variables investigated vary per study. This makes comparison between the studies difficult (Bursac et al., 2010).

Recently two meta-analyzes on stem cell based therapies in (i) chronic ischemic heart disease and congestive heart failure (Fisher et al., 2014) and (ii) acute MI (de Jong et al., 2014) were published. Each study included more than 20 randomized clinical trials adding up to more than 1200 patients. In the first meta-analysis concerning heart failure patients (without MI) there is moderate evidence that stem cells improve LVEF and that it has potential beneficial clinical outcome in terms of performance status (at least 1 year) and mortality (Fisher et al., 2014). The authors do underline the fact that the conclusions are still of low quality as the studies used are small and aimed at safety and feasibility. The second meta-analysis involving cell based therapies in patients with acute MI noted that there was a reasonable improvement in LVEF, which were explained by reduction in scar size and sustained LV end systole volume. Unlike the meta-analysis on heart failure no effects on clinical outcomes were found (de Jong et al., 2014). Similar to the other meta-analysis is that the studies included were only targeted at feasibility and safety.

Not only must cell therapy be delivered in a safe manner and at the right time, the cells should also be capable of making electrotonic connections with CM so that they can integrate and be properly functional within the host's myocardium. Currently, a number of different stem cell types are known, and although these are believed to behave differently from each other, this paper will evaluate studies that have used cardiomyocytes and stem cells, without focusing on one specific type/regardless of the type. The following questions will be addressed: (i) Are stem cells capable of forming functional intercellular connections with cardiomyocytes? and (ii) can pro-arrhythmic effects results from the interaction between stem cells and cardiomyocytes?

## Intercellular connections

Gap junctions are specialized intercellular channels found in the membranes of neighboring cardiomyocytes, and allow exchange of ions and small molecules between cells (Kanno and Saffitz, 2001). These specialized regions are composed of two hexameric hemichannels, one in each neighboring CM. Each hemichannel, or connexon, in turn consists of 6 subunits called connexins (Kanno and Saffitz, 2001; Dhein, 2004). The gap junctional channels allow propagation of the action potential from one cell to the next. In normal healthy cardiomyocytes gap junctions are predominantly found at the intercalated disks, allowing the action potential to spread predominantly along the fiber's axis, resulting in faster conduction along the fiber than across the fiber direction (anisotropy) (Kanno and Saffitz, 2001).

The properties of these gap junctions play an important role in the determination of conduction velocity, and the electrical activation of the heart. Several cardiac connexin isoforms have been identified, each with a different channel conductance; the most predominantly expressed gap junction in ventricular myocytes is connexin43 (Cx43) (van Kempen et al., 1995). The lack of or loss of Cx43 is associated with diminished electrical coupling and formation of arrhythmias (de Groot et al., 2003; de Diego et al., 2008; Procida et al., 2009). Heart failure models show that decreased Cx43 coupling leads to an increase in conduction heterogeneity and to an increase in the inducibility of arrhythmias (Wiegerinck et al., 2008). The relation between uncoupling and conduction velocity is non-linear implying that a relatively large degree of loss of gap junctions is required to cause conduction slowing (Jongsma and Wilders, 2000). Furthermore, reduced intercellular coupling causes increased heterogeneity of repolarization and can contribute to a pro-arrhythmic substrate (Wiegerinck et al., 2008).

Because gap junctions are of crucial importance for the electrical activation and repolarization of the heart, alteration in gap junctions can lead to fundamental changes in the electrical activity of the heart. Stem cells must therefore be not only capable of coupling with CM but also do this with a sufficiently high gap junctional conductance.

## Electrotonic interactions between stem cells and cardiomyocytes

### *In vitro* studies

Various *in vitro* studies employing immunofluorescence staining have shown that gap junctions containing Cx43 exist between mesenchymal stem cells (MSC) and neonatal rat cardiomyocytes (NRCM) (Beeres et al., 2005; Chang et al., 2006; Pijnappels et al., 2006). Beeres et al. and Chang et al. additionally have proved, through the use of dye transfer, that these gap junctions were functional (Beeres et al., 2005; Chang et al., 2006).

The question therefore remains whether the gap junctions between MSC and cardiomyocytes also have (adverse) electrophysiological consequences. Electrophysiological functionality can be confirmed in *in vitro* studies with uncoupling substances such as gap junction blockers. Intercellular uncoupling or blocking of the formation of electrotonic connections can be used to detect both potential pro-arrhythmic as anti- arrhythmic consequences of the interconnections between the cell types. Using Tetrodotoxin in an *in vitro* co-culture of NRCM and human MSC, Askar et al. demonstrated that hetero-cellular coupling was the predominant mechanism for the observed conduction slowing in these preparations (Askar et al., 2013). Beeres et al. showed that electrotonic coupling with human MSC was needed to join two asynchronously beating fields of cardiomyocytes (Beeres et al., 2005). When carbenoxolene, a potent gap junctional uncoupling agent (de Groot et al., 2003), was added to these cultures the synchronized beating of the two fields was lost (Beeres et al., 2005; Pijnappels et al., 2006).

Another approach to establish functionality of connections between CM and stem cells is via genetic modification. In the *in vitro* model used by Beeres and Pijnappels, Cx43 knockdown in human MSC led to failure of synchronization of two asynchronously beating cardiomyocyte fields (Pijnappels et al., 2006). Co-cultures of skeletal myoblasts and NRCM demonstrated a decrease in conduction velocity and easily inducible re-entry, both of which could be attenuated when skeletal myoblasts were modified to overexpress Cx43 (Abraham et al., 2005; Tolmachov et al., 2006; Roell et al., 2007). More efficient coupling therefore appears to be beneficial whereas incomplete coupling is potentially arrhythmogenic.

### *In vivo* studies

Demonstrating functional coupling between stem cells and resident cardiomyocytes in *in vivo* studies is challenging. The implanted cells are usually not directly accessible and the lack of high-resolution functional measurements in a beating heart hampers direct assessment of functional coupling between stem cells and endogenous cardiomyocytes. Post mortem histological analyses may demonstrate successful engraftment of stem cells but not functional coupling.

Autologous stem cells engraftment has also been established in various studies through labeling stem cells with a cross-linkable membrane dye (Dye IL) (Price et al., 2006), transfection with GFP labeled lenti-virus (Valina et al., 2007; Mazo et al., 2011), or transfected *E. Coli* lac Z encoding B-gal (Rigol et al., 2010). Species-specific antibodies can determine the engraftment when xenografted cells are applied (Chong et al., 2014). However, in histological sections the electrophysiological interaction due to functional gap junctions cannot be demonstrated.

Alternative techniques to establish functional coupling *in vivo* is through calcium transients. Rubart et al. transplanted GFP expressing fetal cardiomyocytes into adult mice hearts (Rubart et al., 2003). Imaging of calcium transients in the intact heart of both host and donor cells showed that they were identical, encouraging the concept that cells can functionally couple to host cells (Rubart et al., 2003). Recently, this same concept was used to confirm functional coupling of human embryonic stem cell derived cardiomyocytes in a non-human primate myocardium (Chong et al., 2014). This last study convincingly supported the idea that stem cells can form a functional and synchronized network with the host myocardium. It has to be noted though that the cells used in this study are cardiomyocytes created from embryonic stem cells. This is where stem cell derived cardiomyocytes are purified and cryopreserved before application.

Kehat et al. performed *in vitro* electrophysiological analysis of hES that had formed spontaneous beating embryoid bodies. He demonstrated that hES had both structural and electrical features that resembled those of CM (Kehat et al., 2001). These hES were not grown together with myocytes and therefore it is not possible to state whether electrical coupling would form. Nevertheless, the fact that these cells obtained structural features specific for cardiomyocytes is attractive, because for proper integration within the myocardium the current generated in the host cell must not only pass through gap junctions to depolarize the transplanted cell, these cells must also have the appropriate excitation-contraction coupling properties.

## Pro-arrhythmic effects of electrotonic connections

**Figure 1:**
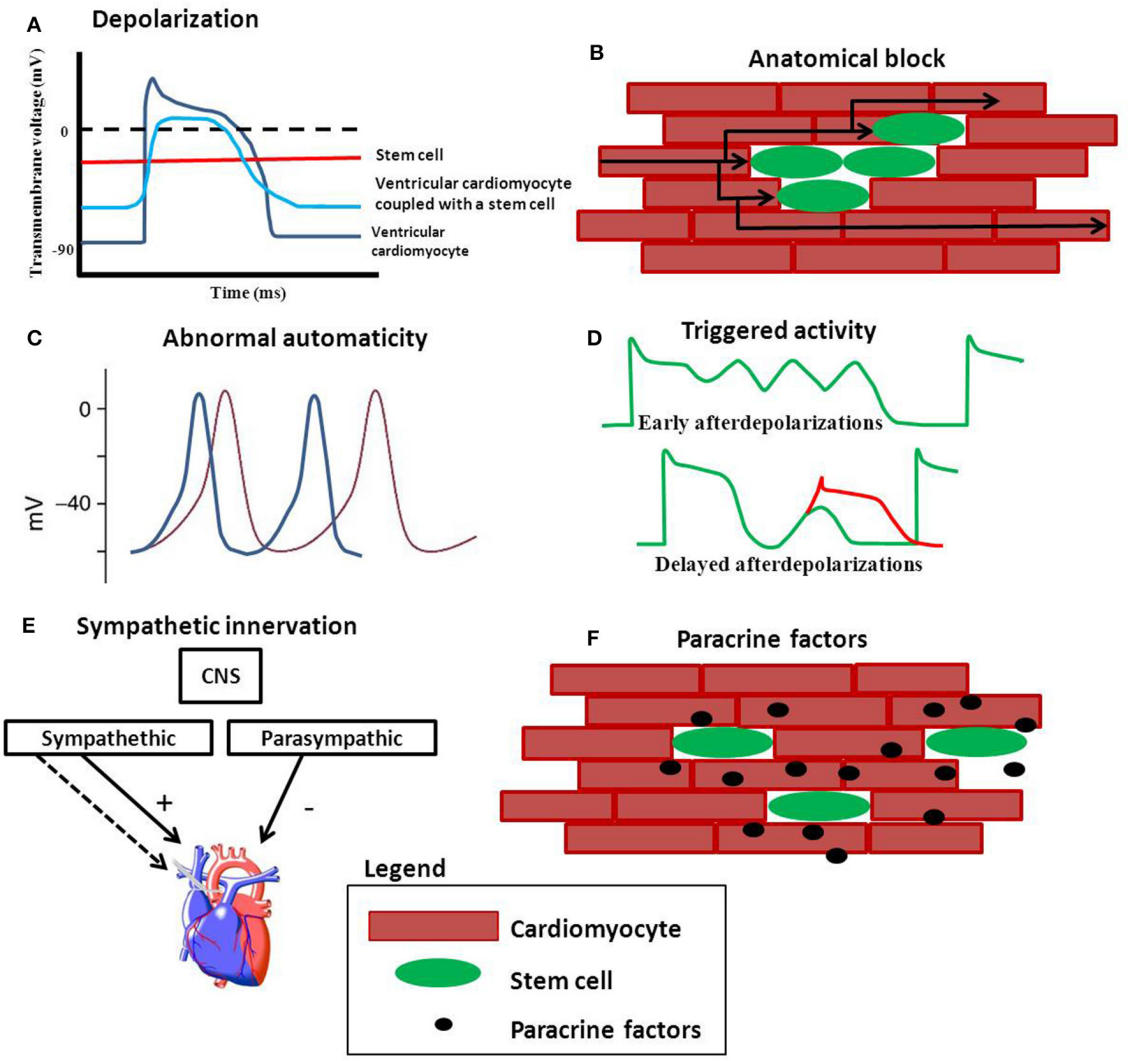
**Possible mechanisms of stem cell induced arrhythmias. (A)** Depolarization of cardiomyocytes reduces the upstroke velocity and conduction velocity. **(B)** Clusters of stem cells can create an anatomical block and force the electrical pathway to find a different (and longer) route. **(C)** Stem cells can be spontaneously beating and may compete with the hosts own automaticity when engrafted. Stem cells may also be capable of inducing arrhythmias via triggered activity **(D)**. **(E)** Increased sympathetic innervation induced by stem cells can give an unbalance in the sympathetic and parasympathetic equilibrium. **(F)** Paracrine factors released by stem cells may effect electrophysiology of cardiomyocytes.

Although engraftment of stem cells may occur, it does not imply that the engrafted cells are functionally and electrically coupled to resident cardiomyocytes. In addition, if these cells do couple electrically it is not self-evident that these interactions can contribute to the synchronized network of endogenous cardiomyocytes without any risks or side effects. It has been suggested that stem cells are capable of influencing the electrophysiological properties of cardiomyocytes, resulting in the formation of a pro-arrhythmic substrate (Chang et al., 2006; Askar et al., 2013). Proposed mechanisms including depolarization, anatomical block, abnormal automaticity and triggered activity are discussed and are schematically shown in Figure 1.

### Depolarization and refractory period

The membrane potential of MSC has been shown to be around −35 mV (Heubach et al., 2004; Valiunas et al., 2004; Sundelacruz et al., 2008). After electrical coupling, depolarization of the resting membrane of CM is anticipated (Xie et al., 2009). The magnitude of depolarization depends on the membrane potential of the connected stem cell, but also to the extent of the intercellular coupling between the two cells (the coupling resistance) and the size of the cells (Tan and Joyner, 1990; Henriquez et al., 2001).

If depolarization of the resting membrane is severe (by more than 30 mV) the excitability of the cells is reduced; the voltage gated sodium channels are inactivated, resulting in reduction in upstroke velocity and conduction velocity (Shaw and Rudy, 1997; Kléber and Rudy, 2004; Hubbard et al., 2007). Also, the plateau phase is expected to be shorter while the action potential duration is expected to be prolonged Figure 1A. Action potential prolongation can lead to (unidirectional) block at tissue level and may facilitate re-entrant arrhythmias (de Bakker et al., 1993; Coronel et al., 2013).

Electrotonic connections between stem cells and cardiomyocytes can also lead to the “current sink” or “current source” phenomenon (Rohr, 2004). This occurs when the load of an excited region supplying the depolarization current is not matched to the amount of current needed to excite the region ahead; current to load mismatch (Rohr, 2004). Stem cells depolarize cardiomyocytes, suggesting that the regions where stem cells are applied are more depolarized and that the depolarizing current coming from these hetero-cellular areas is not enough to excite the regions, consisting of endogenous cardiomyocytes, around it.

Depolarization of cardiomyocytes can thus lead to a reduction in upstroke velocity and conduction velocity but also affect the duration and shape of an action potential. These properties are all related to refractoriness and restitution properties (Hondeghem et al., 2001). Alterations in restitution properties and the effective refractory period (ERP) may lead to arrhythmias (Qu et al., 1999; Tran et al., 2007). The ERP plays an important role, together with conduction slowing and unidirectional block, in facilitating re-entry arrhythmias at a multicellular level. In a porcine MI model administration of MSC shortened ERP and resulted in steeper ERP restitution curves (Price et al., 2006), both indicators of electrical instability.

### Anatomical block

Cells used for cell-based regenerative therapies do not necessarily have to couple functionally to generate potential threatening situations for the formations of arrhythmias. Cells injected into the myocardium can integrate in such a way that they nestle themselves between CM causing resident CM to uncouple or prevent the coupling of CM (Duffy, 2008). Islands of uncoupled cells can cause anatomical block, forcing the electrical wave front to find an alternative route, resulting in a possible increased activation time over a specific region (de Bakker et al., 1993) (Figure 2A). If this results in heterogeneous conduction slowing it could facilitate unidirectional block and re-entrant arrhythmias (Lammers et al., 1990; de Bakker et al., 1993). In infarcted rat myocardium the transplanted myoblasts were functionally isolated from the resident cardiomyocytes (Leobon et al., 2003). Although intracellular recordings in the heart were obtained and the action potentials evoked in the myotubes were accompanied by (local) contractions, these did not spread out over the myocardium (Leobon et al., 2003). Information on conduction heterogeneity and local (fractionated) electrograms were not present in this publication. Both factors could have indicated discontinuous conduction (de Bakker et al., 1993; de Bakker and Wittkampf, 2010) a potential indicator for arrhythmogenesis.

### Abnormal automaticity

Abnormal automaticity is yet another mechanism that can evoke arrhythmias. The normal regular spontaneous sinus rhythm can be lost because other excitable cells have a higher spontaneous activity than the original endogenous pacemaker cells (Figure 1C). This could hold true for human embryonic stem cells that form embryoid bodies of spontaneously beating cardiomyocytes. Kehat et al. transplanted clusters of spontaneously beating cardiomyocytes (derived from hES) into a swine with complete atrio-ventricular block (Kehat et al., 2004). They demonstrated that the ventricular ectopic rhythm that followed the transplantation was localized at the site of cell transplantation. The observation that the transplanted cells were able to pace the entire heart at first glance appears to be positive, especially for bio-pace making purposes (Rosen et al., 2007; Li, 2012). However, the pacing rhythm of the transplanted cells may interfere with the animal's own sinus rhythm. In a recent paper published by Chong et al. human embryonic stem cell derived cardiomyocytes were injected into a non-human primate model of myocardial ischemia and reperfusion. The animals showed an usual high number of non-lethal arrhythmias (Chong et al., 2014). Compared to small animal models (Laflamme et al., 2007; Shiba et al., 2012), in which the same human embryonic cells were also used, the high number of non-lethal arrhythmias seen in the non-primate model may be explained by graft automaticity. These animals have lower heart rates compared to the smaller animals (mice and rats) used prior (Chong et al., 2014).

### Triggered activity

Triggered activity initiated by (delayed or early) after depolarizations, may also be involved in arrhythmogenesis caused by stem cell therapy (Figure 1D).

Although a scarce number of studies concerning human embryonic stem cell derived CM (Jonsson et al., 2011) and induced pluripotent stem cell derived CM (Knollmann, 2013) have been performed to look at pro-arrhythmic potential, no conclusion can be drawn yet to associated stem cells directly with early after depolarizations or delayed after depolarizations.

### Sympathetic Hyper-innervation

Stimulation of sympathetic nerve sprouting is an alternative mechanism of stem cells that promote arrhythmogenesis (Pak et al., 2003; Kim et al., 2010). Although this process does not require the formation of gap junctions it is important to keep in mind when evaluating the safety of stem cells. The heart is innervated by both the parasympathetic and sympathetic nervous system (Figure 1E), whereby the latter has a higher density in de sub-epicardium and central conduction system (Kawano et al., 2003). Sympathetic stimulation leads to acceleration of the heart rate, contractility and can improve cardiac output. However, it has also been shown that disturbed innervation can cause lethal arrhythmias (Cao et al., 2000). Pak et al. stated that MSC induce cardiac nerve sprouting in a large animal model of MI (Pak et al., 2003). One month post MI induction, swines received injections of either a mixture of MSC and bone marrow cells, bone marrow cells alone or only the culture medium. One month later the animals were killed and immunofluorescence staining was performed on tissue samples. Samples were stained for nerve sprouting (growth-associated protein; GAP-43), sympathetic nerves (tyrosine hydroxylase; TH) and for tenascin expression. Animals receiving the combination treatment showed more GAP43-positive nerves, higher density of sympathetic nerves and higher tenascin expression (Pak et al., 2003) suggesting an increase in cardiac (sympathetic) nerve sprouting.

Kim et al. saw similar results when MSCs were injected in the myocardium of canines. In addition, he demonstrated the pro-arrhythmic risk of this hyper-innervation (Kim et al., 2010). Occurrence of ventricular fibrillation was 0% in the sham group and 33.3% in the group who received MSC. Hyper-innervation may therefore be an important mechanism for the pro-arrhythmic potential of stem cells.

### Paracrine factors

An additional mechanism through which stem cells can be pro-arrhythmic is the influence of secreted paracrine factors or factors released once these cells are connected to cardiomyocytes (Figure 1F). Although the “paracrine hypothesis” (Gnecchi et al., 2008) is suggested to be an indirect mechanism behind improvement in left ventricular (LV) function, changes in scar properties, angiogenesis induction and/or improvement in survival of endogenous cardiomyocytes, several studies have documented pro-arrhythmic effects of paracrine factors (Pedrotty et al., 2009; Askar et al., 2013). However, the actual factor(s) and mechanism behind these observed results still need to be identified.

## Conclusion

After nearly two decades of research in stem cell based therapies for cardiac regeneration the search for the “perfect” stem cell—based therapy for cardiac tissue regeneration continues as there are still many questions left that remain unanswered.

Stem cells have the ability to couple electrically to cardiomyocytes via gap junctions. Formations of functional intercellular connections influence the electrical properties of cardiomyocytes and are associated with pro-arrhythmic effects. Depolarization of resting membrane, heterogeneous conduction slowing, and electrical instability are factors that can occur after electrotonic connections between stem cells and cardiomyocytes are formed. Depending on the magnitude of effect these factors can contribute to the formation of arrhythmias.

Future studies should not only be aimed at efficacy and long-term effects but also in making clinical trials similar so that factors (e.g., different in/exclusion criteria, cells dose/type, timing, and route of cell infusion) contributing to conflicting data can be limited and a provide solid framework for the safe application of stem cells without arrhythmogenic side effects.

## Funding

The financial contributions of ICARUS of the BioMedical Materials program (project-P5.01) and the LeDucq (SHAPEHEART network) are gratefully acknowledged.

### Conflict of interest statement

The authors declare that the research was conducted in the absence of any commercial or financial relationships that could be construed as a potential conflict of interest.
